# Association between Clinical Characteristics and Laboratory Findings with Outcome of Hospitalized COVID-19 Patients: A Report from Northeast Iran

**DOI:** 10.1155/2021/5552138

**Published:** 2021-02-05

**Authors:** Sahar Sobhani, Reihaneh Aryan, Elham Kalantari, Salman Soltani, Nafise Malek, Parisa Pirzadeh, Amir Yarahmadi, Atena Aghaee

**Affiliations:** ^1^Department of Nursing and Midwifery, Faculty of Nursing, Gonabad University of Medical Sciences, Gonabad, Iran; ^2^Clinical Research Development Unit, Imam Reza Hospital, Mashhad University of Medical Sciences, Mashhad, Iran; ^3^Assistant Professor of Pulmonology, Department of Pulmonology, Isfahan University of Medical Science, Isfahan, Iran; ^4^Kidney Transplantation Complications Research Center, Mashhad University of Medical Sciences, Mashhad, Iran; ^5^Department of Nutrition Sciences, Varastegan Institute for Medical Sciences, Mashhad, Iran; ^6^Department of Nursing and Midwifery, Faculty of Nursing, Mashhad University of Medical Sciences, Mashhad, Iran; ^7^Transplant Research Center, Shiraz University of Medical Sciences, Shiraz, Iran; ^8^Department of Clinical Biochemistry, Faculty of Medicine, Mashhad University of Medical Sciences, Mashhad, Iran; ^9^Nuclear Medicine Research Center, Mashhad University of Medical Sciences, Mashhad, Iran

## Abstract

Coronavirus disease 2019 (COVID-19) was first discovered in December 2019 in China and has rapidly spread worldwide. Clinical characteristics, laboratory findings, and their association with the outcome of patients with COVID-19 can be decisive in management and early diagnosis. Data were obtained retrospectively from medical records of 397 hospitalized COVID-19 patients between February and May 2020 in Imam Reza Hospital, northeast Iran. Clinical and laboratory features were evaluated among survivors and nonsurvivors. The correlation between variables and duration of hospitalization and admission to the intensive care unit (ICU) was determined. Male sex, age, hospitalization duration, and admission to ICU were significantly related to mortality rate. Headache was a more common feature in patients who survived (*p*=0.017). It was also related to a shorter stay in the hospital (*p*=0.032) as opposed to patients who experienced chest pain (*p*=0.033). Decreased levels of consciousness and dyspnea were statistically more frequent in nonsurvivors (*p*=0.003 and *p*=0.011, respectively). Baseline white blood cell (WBC) count, erythrocyte sedimentation rate (ESR), and C-reactive protein (CRP) were significantly higher in nonsurvivors (*p* < 0.001). Patients with higher WBC and CRP levels were more likely to be admitted to ICU (*p*=0.009 and *p*=0.001, respectively). Evaluating clinical and laboratory features can help clinicians find ways for risk stratifying patients and even make predictive tools. Chest pain, decreased level of consciousness, dyspnea, and increased CRP and WBC levels seem to be the most potent predictors of severe prognosis.

## 1. Introduction

In December 2019, coronavirus disease 2019 (COVID-19) caused by severe acute respiratory syndrome coronavirus (SARS-CoV-2) was first discovered in Wuhan, Hubei Province, China, and has rapidly spread all over the world, causing a pandemic. The virus is considered to be transmitted through respiratory droplets and contaminated surfaces [1–3]. As of October 2020, there are >1 million documented deaths caused by COVID-19 [4]. In Iran, on February 19, 2020, two patients in Qom City were confirmed as COVID-19 positive. Ultimately, the disease spread expeditiously in adjacent provinces near Qom, such as Tehran, Markazi, Isfahan, and Khorasan Razavi provinces and, shortly after, in all 31 provinces of the country [5]. To help better diagnose COVID-19, clinicians and public health professionals should consider the wide variety of signs, symptoms, and laboratory findings of COVID-19 [6].

Although COVID-19 has various clinical manifestations, most patients experience very little or no symptoms, especially in the early disease stage [7, 8]. The incubation period of COVID-19, extending from exposure to onset of disease symptoms, is estimated at approximately 5.2 days [1, 9]. This infection's common signs and symptoms include fever, cough, fatigue, sore throat, chest pain, dyspnea, myalgia, headache, anosmia, ageusia, and diarrhea. In more severe cases, the infection can cause pneumonia, acute respiratory distress syndrome (ARDS), kidney failure, and even death [10, 11].

The current knowledge surrounding clinical characteristics, laboratory findings, and their association in critically ill patients with COVID-19 infection is limited but can prove to be decisive in the management and early diagnosis of this deadly disease.

In this survey, we studied patients with confirmed COVID-19 who were admitted to Imam Reza Hospital of Mashhad, Iran. The data regarding the association between clinical presentations, laboratory findings, and survival of COVID-19 patients will be of considerable value for the early identification of individuals at risk of becoming critically ill and most likely to benefit from intensive care treatment.

## 2. Materials and Methods

### 2.1. Study Population and Data Collection

All patients with confirmed COVID-19 diagnoses admitted to Imam Reza Hospital in northeast Iran between February and May 2020 were enrolled in the study. The COVID-19 infection was confirmed by polymerase chain reaction (PCR) test and lung high-resolution computed tomography (HRCT) results, as instructed in Iranian National Guidelines. The patient outcome was defined as the patient's status at discharge, whether the patient was alive or deceased. All patients were admitted following moderate to severe stages of the disease (i.e., more than 40% of the lung parenchyma affected or instability of vital signs such as hypoxemia and hypotension or severe leukopenia).

In the first hours of admission, all patients were interviewed. A complete medical history, drug history, patient characteristics, laboratory tests, medications prescribed to the patient, paraclinical assessments, and patient outcomes were recorded in the COVID-19 registry of Imam Reza Hospital. Patients were treated according to the national and universal COVID-19 management guidelines.

### 2.2. Ethics Statement

The study protocol was approved by the Ethics Board of Mashhad University of Medical Sciences (IR.MUMS.REC.1399097). Patient's personal information was entered as codes in the database, and the identities of patients were anonymous. The study was carried out under the principles of the Declaration of Helsinki.

### 2.3. Statistical Analysis

Categorical variables were expressed as frequency and percentage. Moreover, continuous variables were expressed as mean ± standard deviation (SD). Chi-squared test, Fisher exact test, and Student's *t*-test were used for nonparametric and parametric analysis, respectively. Pearson's correlation test was used to assess the relationship between clinical features and laboratory findings with hospitalization duration and admission to ICU. A *p* value <0.05 was considered statistically significant. All statistical analyses were performed using SPSS 22 software (SPSS, Chicago, IL, USA).

## 3. Results

A total of 397 patients were included in this analysis. The mean age was 60.6 years (ranging from 14 to 94 years), 223 (56.2%) were males, and 174 (43.8%) were females. 336 patients (84.6%) survived and were discharged in a stable condition, but unfortunately, 61 patients (15.4%) were deceased. The mean duration of hospitalization was 9.3 days, which was significantly longer in the nonsurvivors (*p* < 0.001). The mean age was higher in those who died than those discharged alive (66.01 and 59.64 years, respectively) (*p*=0.013). Males had a higher mortality rate than females (18.8% and 10.9%, *p*=0.035). 68 patients (17.5%) required ICU management, and mortality was significantly higher in these patients (*p* < 0.001) (Table 1).

Common baseline clinical signs/symptoms among our patients were chest tightness (261 patients (65.7%)), fever (225 patients (56.7%)), and myalgia (126 patients (31.7%)). Decreased levels of consciousness and dyspnea were statistically more frequent in nonsurvivors (*p*=0.003 and *p*=0.011, respectively). Interestingly, headache was a more common feature in patients who survived (*p*=0.017). We also investigated three important laboratory findings in all patients: white blood cell (WBC) count, erythrocyte sedimentation rate (ESR), and C-reactive protein (CRP), which were all significantly higher in nonsurvivors (*p* < 0.001) (Table 2).

We also evaluated the correlation between clinical features and laboratory findings with hospitalization duration and admission to ICU. Patients who reported headaches had a shorter stay in the hospital (*p*=0.032) than patients who experienced chest pain, who spent more days hospitalized (*p*=0.033). Moreover, patients with chest tightness were more likely to be admitted to ICU (*p*=0.033) (Table 3). The patients with higher CRP levels had a longer stay in the hospital (*p*=0.006). Furthermore, patients with higher WBC and CRP levels were more likely to be admitted to ICU (*p*=0.009 and *p*=0.001, respectively).

## 4. Discussion

COVID-19 has a range of severity from no symptoms to ARDS. The specific determining factors for the severity of the disease are not well recognized yet. Regarding limited health facilities and a large number of patients referred to hospitals, knowing more about this disease's clinical course helps decide whom to be admitted. In other words, the more we know about the determinants of the clinical course, we can use them in risk stratifying patients and predicting clinical outcomes.

In the present study, the mean age of patients was 60.6 years. A report from the United States in March 2020 showed that 62% of COVID-19 patients were older than 55 (COVID TC). In our study, the mean age of nonsurvivors was significantly higher than those who survived (66.01 and 59.64, respectively). This finding was also noted in some other studies, such as in Italy [12] and the United States [13]. They found older age as a predictor of mortality in patients with COVID-19. Our results showed that the mortality rate was higher in males. Similarly, in a cohort study by Palaidimos et al. in the United States, male sex was associated with worse outcomes [14]. Another study in Wuhan also showed male sex as a risk factor for severity and mortality [15].

WBC may be useful in risk stratifying. In our study, the initial WBC count was in direct correlation with mortality and ICU admission. Unfortunately, we did not include lymphocyte count in this survey. Several studies showed that lymphocyte percentage is inversely associated with the severity of COVID-19 disease [16, 17]. A study by Chen et al. showed that an increased number of neutrophils was a predictor of disease severity [18].

According to our findings, CRP levels were significantly higher in nonsurvivors. Also, hospitalization duration in patients with higher CRP levels was longer, and they were more likely to be admitted to ICU. Moreover, elevated CRP correlation with disease severity has been shown in other studies [19].

Belvis et al. showed that headache was the fifth most frequent symptom after fever, cough, myalgia/fatigue, and dyspnea. In the present study, headache was not a common symptom among our patients (14.9%), but the prevalence of headaches was significantly higher in survivors (*p*=0.017). Furthermore, patients with headaches had a shorter duration of hospitalization than patients who experienced chest pain. Similarly, Trigo et al. showed that headache is an independent predictor of lower mortality risk in COVID-19 patients [20]. In another study, evaluating 179 hospitalized patients with COVID-19, mortality was shown to be higher in patients with headache, although it was not statistically significant in multivariate regression analysis [21].

In a study about the symptoms of COVID-19 patients in the United States, chest pain was among the symptoms reported more commonly after March 8, 2020 (8% before and 35% after). However, a more significant portion of patients in this report was not hospitalized [22].

In our study, chest pain prevalence was not significantly different between survivors and nonsurvivors, but the patients who experienced chest pain spent more days in the hospital. Dyspnea, as a subjective experience of breathing discomfort, has been reported to affect less than 50% of COVID-19 patients and is more common in those who will die than in those who recover [18]. Also, in the present study, dyspnea was more common in nonsurvivors than in the survivor group (*p*=0.011), keeping in mind that dyspnea seems to be underreported in patients with COVID-19, which is called happy hypoxemia [23].

A decreased level of consciousness is caused by a variety of etiologies, from hypoxia to neurologic complications. In our study, a decreased level of consciousness, regardless of its etiology, was more common in nonsurvivors. Impaired consciousness has been reported in 7.5% of hospitalized patients with COVID-19, and it has been more likely to be presented in severely affected patients [24]. Similarly, Lahiri and Ardila reported that impaired consciousness was considered among the presenting features of COVID-19 [25].

## 5. Conclusions

The COVID- 19 pandemic poses many challenges, and it is essential to gather as much knowledge as possible about this disease. Clinical characteristics, laboratory findings, and their association with the outcome of patients with COVID-19 can be decisive in the management and early diagnosis. Besides, considering the importance of optimized use of limited health facilities, it is essential to know how to stratify patients and even make predictive tools for better treatment. Chest pain, decreased level of consciousness, dyspnea, and increased CRP and WBC levels seem to be the most potent predictors of severe prognosis.

## Figures and Tables

**Table 1:** Characteristics of survivors and nonsurvivors of COVID-19.

	Total (*n* = 397)	Survivors (*n* = 336)	Nonsurvivors (*n* = 61)	*P* value
Age (years)	60.6 ±17.75	59.64 ± 18.13	66.01 ± 15.04	0.013
Sex (male/female)	223/174	180/156	42/19	0.035
Hospitalization (days)	9.35 ± 9.65	8.02 ± 6.8	16.63 ± 16.9	<0.001
Admission to ICU (%)	68 (17.5)	33 (10)	35 (58.3)	<0.001

Data are presented as means ± SDs. ICU: intensive care unit.

**Table 2:** Baseline clinical manifestations and laboratory findings of survivors and nonsurvivors of COVID-19.

	Total (*n* = 397)	Survivors (*n* = 336)	Nonsurvivors (*n* = 61)	*P* value
Fever (%)	225 (56.7)	189 (56.3)	32 (52.4)	0.573
Headache (%)	59 (14.9)	56 (16.7)	3 (5)	0.017
Sore throat (%)	43 (10.8)	36 (10.7)	7 (11.4)	0.825
Chest pain (%)	56 (14.1)	51 (15.2)	5 (8.2)	0.166
Myalgia (%)	126 (31.7)	110 (32.7)	16 (26.2)	0.369
Smell disorder (%)	29 (7.3)	27 (8)	2 (3.3)	0.284
Taste disorder (%)	24 (6)	22 (6.5)	2 (3.3)	0.557
Pharyngeal exudates (%)	7 (1.8)	6 (1.8)	1 (1.6)	0.431
Decreased consciousness (%)	8 (2)	3 (0.9)	5 (8.2)	0.003
Dyspnea (%)	74 (18.6)	55 (16.4)	19 (31.1)	0.011
Chest tightness (%)	261 (65.7)	211 (62.8)	43 (70.5)	0.303
Nausea and vomiting (%)	99 (24.9)	87 (25.9)	11 (18)	0.200
Diarrhea (%)	51 (12.8)	46 (13.7)	5 (8.2)	0.300
Initial WBC (10^3^/mm^3^)	8.80 ± 5.29	8.25 ± 4.51	11.67 ± 7.67	<0.001
Initial CRP (*μ*g/dL)	90.13 ± 77.73	78.86 ± 66.80	140.94 ± 100.92	<0.001
Initial ESR (mm/h)	51.55 ± 32.46	50.34 ± 32.51	57.03 ± 32.03	<0.001

Data are presented as % and means ± SDs. WBC: white blood cells; CRP: C-reactive protein; ESR: erythrocyte sedimentation rate.

**Table 3:** Pearson's correlation coefficients between clinical manifestations and laboratory findings with hospitalization duration and admission to ICU.

	Duration of hospitalization		Admission to ICU	
	*r* values	*P* values	*r* values	*P* values
Fever	0.035	0.489	−0.009	0.865
Headache	−0.109	0.032^*∗*^	0.081	0.109
Sore throat	0.013	0.794	−0.033	0.520
Chest pain	0.108	0.033^*∗*^	−0.015	0.765
Myalgia	0.001	0.983	0.043	0.397
Smell disorder	0.027	0.597	−0.002	0.972
Taste disorder	0.043	0.403	−0.034	0.508
Pharyngeal exudates	0.0005	0.996	0.040	0.437
Decreased consciousness	-0.008	0.875	0.089	0.078
Dyspnea	0.037	0.462	0.070	0.168
Chest tightness	0.021	0.679	0.108	0.033^*∗*^
Nausea and vomiting	−0.029	0 .568	0.018	0.729
Diarrhea	−0.013	0.791	0.042	0.411
Initial WBC (10^3^/mm^3^)	−0.002	0.975	0.139	0.009^*∗∗*^
Initial CRP (*μ*g/dL)	0.16	0.006^*∗∗*^	0.213	0.001^*∗∗*^
Initial ESR (mm/h)	0.045	0.454	0.073	0.228

Data are presented as means ± SDs. ^*∗*^Correlation is significant at the 0.05 level. ^*∗∗*^Correlation is significant at the 0.01 level. WBC: white blood cells; CRP: C-reactive protein; ESR: erythrocyte sedimentation rate.

## Data Availability

Data are available on request to the corresponding author.
